# Mantle Cell Lymphoma Presenting with Acute Bilateral Ophthalmoplegia

**DOI:** 10.4274/tjo.80557

**Published:** 2017-08-15

**Authors:** Yaran Koban, Hatice Özlece, Orhan Ayar, Mustafa Koç, Hüseyin Çelik, Zeliha Yazar, Ayşe Burcu

**Affiliations:** 1 Kafkas University Faculty of Medicine, Department of Ophthalmology, Kars, Turkey; 2 Edirne State Hospital, Neurology Clinic, Edirne, Turkey; 3 Bulent Ecevit University Faculty of Medicine, Department of Ophthalmology, Zonguldak, Turkey; 4 Ulucanlar Training and Research Hospital, Ophthalmology Clinic, Ankara, Turkey

**Keywords:** mantle cell lymphoma, ophthalmoplegia, blepharoptosis

## Abstract

A 72-year-old woman presented with acute onset of double vision, bilateral complete blepharoptosis, and nearly complete ophthalmoplegia. Orbital and brain magnetic resonance imaging were normal. Further investigation revealed bicytopenia with hepatosplenomegaly. Liver biopsy revealed mantle cell lymphoma. Cytology later showed the presence of mantle cells in cerebrospinal fluid analysis. Her ophthalmoplegia improved from her first cycle of systemic and intrathecal chemotherapy. To the best of our knowledge, this is the second case in the literature of mantle cell lymphoma with central nervous system involvement presenting with ophthalmoplegia. This symptom should be considered one of the initial signs of mantle cell lymphoma.

## INTRODUCTION

Mantle cell lymphoma (MCL) comprises 5% of non-Hodgkin lymphoma. In general, patients are typically Caucasian (about 2:1), male (about 2.5:1), and elderly (median age of onset, 68 years), and they usually present with extensive disease, including widespread lymphadenopathy, bone marrow involvement, splenomegaly, circulating tumor cells, and bowel infiltration.^1^ Central nervous system (CNS) involvement is an unusual form of extranodal involvement in the course of MCL. We present a rare case of MCL with CNS involvement with ophthalmoplegia and negative imaging studies. To the best of our knowledge, this is the second reported case in the literature.

## CASE REPORT

**Figure 1:**
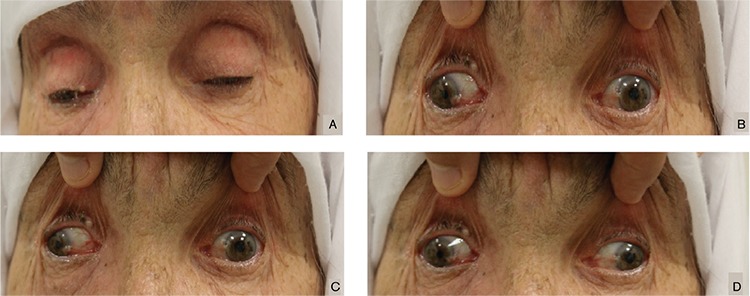
A) Bilateral complete ptosis and B) right exotropia in primary gaze position; C, D) Nearly complete ophthalmoplegia in both eyes except minimal abduction

A 72-year-old woman presented with a 1-week history of progressive blepharoptosis and diplopia. On examination, she had bilateral complete blepharoptosis and right exotropia in primary gaze position. There was nearly complete ophthalmoplegia in both eyes except minimal abduction (Figure 1). The right pupil was 2 mm and reacted sluggishly to direct light. The left pupil was 4 mm and nonreactive. Assessment of motility revealed noticeable underaction of both superior oblique muscles. Corneal sensation was intact bilaterally. Visual acuity was 20/20 in both eyes. All other aspects of the ophthalmologic and neurologic examinations were normal. Orbital and cranial computerized tomography were also normal. Her past medical history and family history were unremarkable.

Following an evident loss of weight estimated to be about five kilograms in three months, the attending physician requested a blood test which revealed deterioration in the liver function tests and bicytopenia. Computerized tomography scan of the chest, abdomen, and pelvis revealed hepatosplenomegaly. There were interstitial changes in the lung bases along with left pleural effusion. There was no lymphadenopathy. A magnetic resonance imaging (MRI) study of the brain was unremarkable.

The liver biopsy revealed diffuse infiltration by a MCL (CD20+, CD5+ and cycline D1+). She was then referred to the hematology department.

CNS invasion of MCL was suspected on the basis of clinical features, but no abnormalities were detected in serial contrast-enhanced MRI studies. Lumbar puncture revealed normal opening pressure and showed exaggerated lymphocytic pleocytosis, a protein level of 174 mg/dL, and glucose level of 51 mg/dL. Cytology later showed the presence of mantle cells in cerebrospinal fluid analysis. Combined systemic and intrathecal chemotherapy with rituximab, cyclophosphamide, doxorubicin, vincristine and prednisone (R-CHOP) was administered for eight cycles in parallel with intrathecal injections of methotrexate and cytarabine. After the first cycle, her ophthalmoplegia and blepharoptosis improved. Recurrent ophthalmoplegia and blepharoptosis were not observed during the treatment process. The patient was followed by the internal medicine department and was referred to a tertiary cancer center for further treatment.

## DISCUSSION

MCL is a very aggressive subtype of non-Hodgkin lymphoma and is unique among lymphomas in its clinical, biologic, and genetic properties. Nearly 70% of cases are diagnosed in advanced stages of the disease and most cases exhibit a relatively aggressive course. Median life expectancy ranges from 3 to 7 years. Because of its unresponsiveness to medical treatment as well as its aggressive nature, MCL is generally considered incurable.^2^

MCL usually involves the lymph nodes, spleen, and bone marrow. Extranodal involvement is often seen in the gastrointestinal tract and Waldeyer’s ring. In most cases, the abovementioned organs are diffusely involved and the disease is generally diagnosed in later stages. The disease may also affect the breasts, lungs, soft tissues, salivary glands, and orbit. CNS involvement is seen mostly in recurrent disease and is rare at first presentation.^3^

Cheah et al.^4^ presented the largest series of patients with MCL and CNS involvement reported to date. This study showed that the crude incidence of CNS involvement was 4.1%, with 0.9% having CNS involvement at diagnosis. The most frequent clinical manifestations of CNS involvement included signs and symptoms related to high intracranial pressure or meningeal infiltration, and mainly consisted of mental status changes, headache, cranial nerve palsies and diplopia. Symptoms at presentation varied, but they noted ocular disturbance in 20% of 57 patients with MCL and CNS involvement.^4,5^

We report the case of a 72-year-old woman with MCL having partial bilateral third, fourth, and sixth nerve palsy. Although there have been some rare cases reported with blepharoptosis and restricted eye movements as symptoms of non-Hodgkin lymphoma, to the best of our knowledge, ours is the first case of MCL presenting with bilateral ophthalmoplegia and blepharoptosis and the second case in the literature that has shown MCL with CNS involvement manifesting with ophthalmoplegia and negative imaging studies.^6,7,8^

Then and Patel^8^ presented a unique case of a 65-year-old woman diagnosed with stage 4A Kappa restricted B Cell MCL who presented with acute-onset double vision, skew deviation of the eyes, left eye ptosis, right horizontal gaze palsy, right facial droop, dysarthria, and dysphagia 2 months after the lymphoma diagnosis. Orbit computerized tomography and brain MRI were normal. However, as in our case, lumbar puncture showed exaggerated lymphocytic pleocytosis and cytology showed the presence of mantle cells on cerebrospinal fluid analysis.

MRI is the best way to investigate the degree of CNS infiltration, whether intraparenchymatous or meningeal.^9^ Ophthalmoplegia with normal MRI may occur via paraneoplastic encephalomyelitis and leptomeningeal metastasis (lymphomatous meningitis).^10,11^

Neoplastic meningitis, a particular manifestation of CNS recurrence, results from the infiltration of metastatic cells into the cerebrospinal fluid and meninges. Neoplastic meningitis is also referred to as lymphomatous meningitis in patients with lymphoma. Lymphomatous meningitis symptoms can reflect involvement at any level of the neuroaxis, which consists of the meninges (the three-layered sheath enclosing the organs of the nervous system), brain, spinal cord, and cerebrospinal fluid.^12,13^ The analysis of cerebrospinal fluid has made it possible to confirm CNS infiltration. Cerebrospinal fluid cytology is positive in 86% of MCL patients with CNS involvement, and flow cytometry is positive in 91%.^4,14^

Common causes of acute complete bilateral ophthalmoplegia include Miller Fisher syndrome, Guillain-Barre syndrome, posterior-circulation (brainstem) stroke, myasthenia gravis, drug toxicity (e.g. phenytoin), and trauma.^15^ MCL also should be added to the causes of rapidly progressive bilateral ophthalmoplegia. As in other B-cell lymphoma cases, improvement after chemotherapy suggests that early treatment with chemotherapy may effectively treat ophthalmoplegia associated with MCL.
